# Investigation of Encephalopathy Caused by Shiga Toxin 2c-Producing *Escherichia coli* Infection in Mice

**DOI:** 10.1371/journal.pone.0058959

**Published:** 2013-03-13

**Authors:** Muhammad Yunus Amran, Jun Fujii, Satoshi O. Suzuki, Glynis L. Kolling, Sharon Y. A. M. Villanueva, Mosaburo Kainuma, Hideyuki Kobayashi, Hideko Kameyama, Shin-ichi Yoshida

**Affiliations:** 1 Department of Bacteriology, Graduate School of Medical Sciences, Kyushu University, Fukuoka, Japan; 2 Department of Neuropathology, Graduate School of Medical Sciences, Kyushu University, Fukuoka, Japan; 3 Center for Global Health, University of Virginia, Charlottesville, Virginia, United States of America; 4 Department of General Internal Medicine, Kyushu University Hospital, Fukuoka, Japan; 5 Department of Chemistry, School of Medicine, University of Occupational and Environmental Health, Kitakyushu, Japan; 6 Department of Neurology, Hasanuddin University Hospital, Faculty of Medicine, Hasanuddin University, Makassar, South Sulawesi, Indonesia; University of Padova, Italy

## Abstract

A large outbreak of Shiga toxin (Stx)-producing enteroaggregative *Escherichia coli* (EAEC) O104:H4 occurred in northern Germany. From this outbreak, at least 900 patients developed hemolytic uremic syndrome (HUS), resulting in more than 50 deaths. Thirty percent of the HUS patients showed encephalopathy. We previously established a mouse model with encephalopathy associated with blood brain barrier (BBB) damage after oral infection with the Shiga toxin (Stx) 2c-producing *Escherichia coli* O157: H- strain E32511 (E32511). In this model, we detected high expression of the Stx receptor synthase enzyme, glycosphingolipid globotriaosylceramide (Gb3) synthase, in endothelial cells (ECs) and neurons in the reticular formation of the medulla oblongata by *in situ* hybridization. Caspase-3 was activated in neurons in the reticular formation of the medulla oblongata and the anterior horn of the spinal cord. Astrocytes (ASTs) were activated in the medulla oblongata and spinal cord, and a decrease in aquaporin 4 around the ECs suggested that BBB integrity was compromised directly by Stx2c or through the activation of ASTs. We also report the effectiveness of azithromycin (AZM) in our model. Moreover, AZM strongly inhibited the release of Stx2c from E32511 *in vitro*.

## Introduction

A large outbreak of Shiga toxin (Stx)-producing enteroaggregative *Escherichia coli* (EAEC) O104:H4 occurred in northern Germany in May 2011, resulting in at least 4,000 infections reported in 16 countries across Europe and North America [1], [2]. Of these cases, more than 900 developed hemolytic uremic syndrome (HUS), resulting in more than 54 deaths. In the German outbreak, 30% of HUS patients showed signs and symptoms of encephalopathy, including delirium, stimulus-sensitive myoclonus, aphasia, and epileptic seizures requiring mechanical ventilation [3]. These patients were treated with IgG, and IgG complexes were depleted by immunoadsorption [3]. This treatment was effective in improving the severe neurological complications. In Japan, between late April and early May 2011, an outbreak of enterohemorrhagic *E. coli* (EHEC) O111 infection occurred mainly in the Toyama, Fukui prefecture and Yokohama City. A total of 169 people suffered from diarrhea, 30 (17.8%) developed HUS, and surprisingly, encephalopathy developed in 14 (47%) of the HUS patients. Furthermore, 5 hospitalized patients died with neurological manifestations, including somnolence, coma, and convulsions [4].

Central nervous system (CNS) dysfunction is an important predictive factor for HUS and mortality in children [5]–[7]. In 1994, we established a mouse model of CNS disorders by oral infection with the Stx2c-producing EHEC O157: H- strain E32511/HSC (E32511), resulting in damage to the endothelial cells (ECs) of capillaries and to nerve fibers in the cerebral cortex and spinal cord in the mice [8]. In this model, we succeeded in detecting Stx2c in the nerve fibers of the spinal cord by immunohistochemical electron microscopic examination. In another model, Stx2-induced brain edema detected by magnetic resonance imaging (MRI) in rabbits was considered a manifestation of the deterioration of the blood-brain barrier (BBB); Stx2 immunoreactivity was detected on the apical plasma membrane of the endothelial cells in the hypothalamic lesion [9]. Steinborn *et al*. also reported that 100% of six patients with post-diarrhea HUS accompanied by serious CNS involvement had basal ganglia lesions detected by MRI [10]. In the Stx2-treated rabbit model of acute encephalopathy, MRI showed high intensity areas in the spinal cord and medulla oblongata. Based on the analysis of the relation between mean arterial pressure and renal sympathetic nerve activity, the cause of death in the rabbit model was circulatory failure caused by impairment of the cardiovascular center in the medulla [11].

EHEC has two major types of toxins, Stx1 and Stx2, which are comprised of 1A and 5B protein subunits [12]. The A subunit has *N*-glycosidase activity that removes adenine 4324 of 28S RNA of the 60S ribosomal subunit [13]. The amino acid sequence identity between the A subunits of Stx1 and Stx2 has been reported to be only 55% [14]. On the other hand, Stx produced by *Shigella dysenteriae* is identical to Stx1 [15]. Stx2 had an LD_50_ approximately 400 times lower than that of Stx1 when injected intravenously (i.v.) or intraperitoneally (i.p.) into mice [16]. In a baboon model, i.v. administration of Stx2 (25 ng/kg, 12 h each, 4 times) caused HUS, while administration of the same amount of Stx1 did not [17]. Each B subunit binds with high affinity to the glycosphingolipid globotriaosylceramide (Gb3), which is present in some eukaryotic cells [18]. It has been demonstrated that Gb3 (CD77) is up-regulated by lipopolysaccharide (LPS), TNF-α, and IL-1β [19], [20]. CD77 is a marker of human germinal centre B cells [21]. The life-threatening CNS manifestations mediated by Stxs are associated with cell death of human brain microvascular endothelial cells (HBMEC). However, the type of Stx that induces cell death related to loss of 28S RNA is still unclear. Bauwens et al. reported that Stx2 caused apoptosis in HBMECs and EA.Hy923 human macrovascular ECs, but Stx1 only caused necrosis in EA.Hy923 macrovascular ECs [22]. We previously reported that induction of apoptosis by Stx1 in HeLa cells requires caspase-6, -8, and -9 within 4 h, followed by activation of caspase-3, which leads to fragmentation of nuclear DNA [23]. We also reported that Stx2 treatment induced a marked up-regulation of C/EBP homologue protein/growth arrest (CHOP) genes, resulting in endoplasmic reticulum stress (ER stress) [24]. In this study, we performed *in situ* hybridization (ISH) using a Gb3 synthase probe in mouse brain. Previously, we reported that a disruption of BBB integrity was detected in the E32511 model (see Materials and Methods) [8]. However, the mechanism of the disruption of the BBB is not clear; because Gb3 was not detected in ECs of control mice [25]. Recent reports showed that Stx1- and LPS-induced astrocyte (AST) responses could influence the integrity of brain ECs and BBB dysfunction *in vitro* [26]. It is not yet known whether microgliosis is associated with the pathogenesis of the brain impairment. The water channel aquaporin 4 (AQP4) is located in the perivascular end-feet of astrocytes (ASTs) in the brain. An increase in glial fibrillary acidic protein (GFAP) reactivity in perivascular ASTs caused a down-regulation of AQP4 in rats injected with supernatant from a culture of recombinant *E. coli* expressing Stx2 [27].

No treatment has been established for EHEC-related HUS and encephalopathy. In 1996, a large outbreak of EHEC O157:H7 infection occurred among schoolchildren in Sakai City, Osaka, Japan. As many as 10,000 patients suffered from diarrhea and hemorrhagic colitis, 150 patients developed HUS, and 3 children died. After the outbreak in Japan, the treatment guidelines for EHEC infection written by the Ministry of Public Welfare recommended administration of antimicrobial agents, especially fosfomycin (FOM), kanamycin (KM), and norfloxacin (NFLX). As the rationale for this opinion, it was suggested that the administration of chemotherapy at the earliest point of the EHEC infection removes the bacteria and lowers the level of Stxs in the colon. Also, from surveys of physicians who treated EHEC patients, FOM was the most frequently administered, and the authors emphasized that administration within the first two days of illness reduced the risk of HUS [28]. We previously reported an evaluation of the therapeutic effects of the antimicrobial agents FOM, minocycline (MINO), KM and NFLX in our EHEC E32511-infected mouse model [8]. The mortality rate of mice treated with MINO, KM and NFLX was significantly lower than that of the control group, but FOM was not effective [29]. Recently, we discovered an effective therapy in the Stx2 toxemic rabbit. A steroid pulse therapy of betamethasone sodium phosphate (BSP) injected twice a day for 2 days statistically reduced the mortality rate and improved the survival period of Stx2 toxemic rabbits [30]. In a study by Ohara et al, immature mice was the animal model used [31]. Results from Ohara et al`s study showed that azithromycin (AZM) administered i.p. at higher concentrations (10 mg/kg) and three times (24, 48, and 78 h) after STEC inoculation prevented the death of these mice compared with ciprofloxacin (CPFX) treatment group. Moreover, they reported that AZM was effective to reduce the production of the cytokine level such as TNF-α, IL-1β, and IL-6 in Stx-treated monocytes and in serum of Stx-injected mice. In our study, we confirmed the effectiveness of AZM in our E32511 model that showed neurological manifestations and fatal acute encephalopathy after oral infection with 10^11^ CFU/mouse of E32511 and simultaneous i.p. injection with mitomycin C (MMC).

In the current study, apoptosis accompanied by caspase-3 activation was detected in the motor neurons in the anterior horn of the spinal cord and in neurons in the reticular formation of the medulla oblongata. Additionally, GFAP-immunopositive reactive ASTs were observed around the blood vessels and neurons in the E32511 model. Gb3 synthase mRNA was up-regulated in those lesions as a response to the oral inoculation of E32511 or an i.p. injection of LPS. Our mouse model may recapitulate the CNS pathology of E32511 strain, represented by the expression of Gb3, apoptosis in neurons, and disruption of the BBB associated with reactive ASTs.

## Materials and Methods

### Ethics statement

Animal experiments were carried out in strict accordance with the recommendations in Guidelines for Proper Conduct of Animal Experiments of the Science Council of Japan. The protocol was approved by the Ethics Committee on Animal Experiments of Kyushu University, Japan (Permit Number: A23-141-1). All surgical procedures were performed under sevoflurane anesthesia, and all efforts were made to minimize suffering.

### Bacterial strains

E32511 was used in this study [8]. Streptomycin (SM)- and Mitomycin C (MMC)-resistant E32511 were grown in nutrient agar supplemented with 100 µg/mL of SM (Wako Pure Chemical Industries, Ltd., Osaka, Japan), 0.5 µg/mL of MMC (Kyowa Hakko Kogyo Co. Ltd., Tokyo, Japan). *E. coli* K-12, which is resistant to SM and MMC, was used as a negative control. E32511/HSC and *E. coli* K-12 were grown with shaking for 17 h in Luria-Bertani broth (LB broth) at 37°C. The bacterial pellets were then harvested by centrifugation at 6000×*g* for 25 min at 4°C, before being washed twice with phosphate-buffer saline (PBS) by centrifugation (6000×*g* for 20 min at 4°C). Finally, bacterial pellets were suspended in PBS and the appropriate number of CFUs was given orally to the mice.

### Shiga toxin 2 (Stx2)

Purified Stx2 was used and was determined to be free of detectable LPS by the Toxicolor test (Seikagaku Kogyo Co., Tokyo, Japan), sodium dodecyl sufate-polyacrylamide gel electrophoresis, and silver staining.

### Animals

Four week old female ICR outbred mice purchased from Japan SLC, Inc. (Shizuoka, Japan) were used for all *in vivo* experiments in this study. They were housed in cages with a 12 h light-dark cycle and free access to laboratory food and distilled water.

### Mouse infection model of acute encephalopathy

For the *in vivo* experiments, the mice were divided into groups of 5 or 6. In this study, the mice were given E32511/HSC orally and MMC i.p. [8]. Briefly, all mice were given SM-containing drinking water (5 g/L) *ad libitum* to reduce the level of their normal intestinal flora [32]. On day 3 of SM treatment, all the mice were completely deprived of food for 12 h until bacterial inoculation. Each mouse was infected with 0.5 mL of bacterial suspension using a sterile disposable feeding needle (diameter, 1.9 mm; length, 38 mm, Fuchigami Co. Ltd. Nara, Japan) attached to a syringe passed into the stomach by orogastric inoculation. Simultaneously, they were injected i.p. with 2.5 µg/g MMC. We reported that 90% of the mice infected with 5×10^10^ CFU of E32511 orally plus i.p. injection of MMC developed weight loss, flaccid paralysis and tremor, culminating in death [8]. In this study, the bacterial suspension of E32511 was adjusted to 1×10^11^ CFU in PBS and caused 100% death. After the completion of treatment, they were housed according to the treatment groups and given food and SM-containing drinking water (5 g/L) *ad libitum*. The mice were observed twice daily for 2 weeks, for the survival or death of mice and for signs of illness, and measurement of body weight (BW).

### Preparation of mouse brain and spinal cord tissues

The mouse brain and spinal cord tissue were harvested immediately after the mice were anesthetized. Thereafter, mice were perfused with 10 mL of 1× PBS solution through the left cardiac ventricle followed by 10 mL of 4% paraformaldehyde (PFA) (Wako Laboratory Chemicals, Osaka, Japan)/PBS to fix the organ tissue. Brain and spinal cord were harvested and fixed in 4% PFA/PBS. Then, the brain and spinal cord were processed for immunohistochemical examination.

### *In situ* hybridization (ISH) of Gb3-synthase in mouse brain

For detection of Alpha 1,4-galactosyltransferase (Gb3 synthase; Accession # NM_001170954) mRNA, a 532-bp DNA fragment corresponding to nucleotide positions 155-686 of mouse Gb3 synthase was sub-cloned into pGEMT-Easy vector (Promega, Madison, WI, USA) and used to generate sense or antisense RNA probes. We hybridized paraffin-embedded spinal cord sections (6 mm) with digoxigenin-labeled RNA probes at 60°C for 16 h. The bound label was detected using NBT-BCIP (Sigma-Aldrich), an alkaline phosphate color substrate. Sections were counterstained with Kernechtrot stain solution (Muto Pure Chemicals Co., Ltd. Tokyo, Japan). Stained slides were examined using a Keyence (Osaka, Japan) HS All-in-one Fluorescence Microscope BZ-9000E.

### Immunohistochemistry

Formalin-fixed, paraffin-embedded tissue blocks were cut into 3 µm thick sections. The sections (except for those to be stained for GFAP, AQP4, and Iba-1) were deparaffinized in xylene, dehydrated in an ethanol gradient and pretreated for heat-based antigen retrieval with 10 nmol/L citrate buffer solution (pH 6.0) for 20 min in an autoclave. The sections were incubated with 5% nonfat milk to eliminate nonspecific binding of the antibody (Ab), and were then incubated overnight at 4°C with the polyclonal anti-cleaved caspase-3 Ab (1∶400, Cell Signaling Technology, Inc, Beverly, MA, USA) diluted with PBS containing 5% BSA and 0.1% Tween 20. After endogenous peroxidase activity was blocked with methanol containing 1% H_2_O_2_, the sections were incubated with an HRP-labeled anti-rabbit secondary antibody, HistoFine (Dako, Glostrup, Denmark) for 30 min. The target proteins were visualized with the peroxidase stain DAB kit (Nacalai Tesque, Kyoto, Japan). Nuclei were counterstained with hematoxylin. For the immunostaining of astrocytes and AQP4, polyclonal rabbit anti-GFAP (1∶1000; Dako Cytomation, Glostrup, Denmark) and polyclonal rabbit anti-AQP4 (1∶500; Santa Cruz Biotechnology, Santa Cruz, CA, USA) Abs were used [33]. For the immunostaining of microglial activation, a rabbit polyclonal antibody anti-Iba1 Ab (1∶500; Wako Pure Chemical Industries Ltd.) was used and diluted in PBS containing 5% BSA and 0.1% Tween 20 (final concentration 1 µg/mL). Specimens were examined using a Keyence HS All-in-one Fluorescence Microscope BZ-9000E.

### Three-dimensional (3D) images

Serial images of mice spinal cord were captured with a Keyence HS All-in-one Fluorescence Microscope BZ-9000E at 20× magnification. Thereafter, using the serial images, the 3D reconstruction was generated using Neurolucida software (Micro Bright Field Japan, Inc., Chiba, Japan).

### Preparation of AZM, other antimicrobial agents and MIC against strain E32511

AZM was obtained from Pfizer, NY, USA; FOM and KM was purchased from Meiji Seika Kaisha, Ltd., Tokyo, Japan. NFLX, OFLX and CPFX were purchased from Sigma-Aldrich Co., Tokyo, Japan. FOM was diluted with distilled water, and AZM was prepared as a stock solution and dissolved with 50 mM citrate buffer, pH 5.3. NFLX and OFLX were diluted with 0.1 N NaOH at 1 mg/mL as a stock solution and diluted with distilled water. CPFX was diluted with 0.1 N HCl at 1 mg/mL as a stock solution and diluted with distilled water.

The Minimal Inhibitory Concentration (MIC) of each antibiotic against E32511/HSC were determined using a Broth Dilution Method according to the recommendations of the National Committee for Clinical Laboratory Standards (NCCLS 1993) except for FOM, the MIC of which was determined using the agar dilution method [34]. The range of antimicrobial agent concentrations for E32511/HSC were : AZM 512–0.125 µg/mL; FOM 12.48–0.195 µg/mL; KM 100–1.5625 µg/mL; NFLX 0.784–0.0245 µg/mL; OFLX 1.6–0.00625 µg/mL; and, CPFX 0.96–0.00375 µg/mL. The bacterial cultures containing 2-fold serially diluted antibiotics were incubated in a 37°C shaker with aeration for 18 h. The MIC of each antibiotic was obtained by determining the highest dilution or lowest dose of each antibiotic that inhibited the growth of bacteria.

### Bacterial count and concentration of Stx2c

The effect of AZM and other antimicrobial agents *in vitro* was assayed at sub-minimum inhibitory concentrations. Samples were obtained from the MIC examination. Briefly, after 18 h incubation, the bacterial cultures with 0.5× MIC of each antibiotic 2-fold serially diluted were plated on Luria Bertani (LB) agar plates and the bacterial colonies counted, after which the bacterial cultures were collected and centrifuged at 9500×*g*, 4°C for 15 minutes. Culture supernatant was collected and filtered through a 0.22 µM filter before determining the Stx2c level in the culture supernatant using a Reverse Passive Latex Agglutination (RPLA) test kit (Denka Seiken Co.).

### Statistical Analysis

Data were analyzed with SPSS Statistics 19.0 (IBM Japan, Tokyo, Japan). The significance of the survival rate from each treatment group was compared using Kaplan-Meier test followed by a pair-wise comparison of Log Rank (Mantel-Cox) test. Student's t-test was used to show the significant values between negative control, positive control and treatment groups. Spearman's test was used to show the correlation between the dose of AZM and the increase in BW of E32511-infected mice. Significance was set at a value of *p*<0.05 in all cases.

## Results

### Localization of Gb3 synthase mRNA by ISH in the E32511 model and LPS-injected mouse

**Figure 1:**
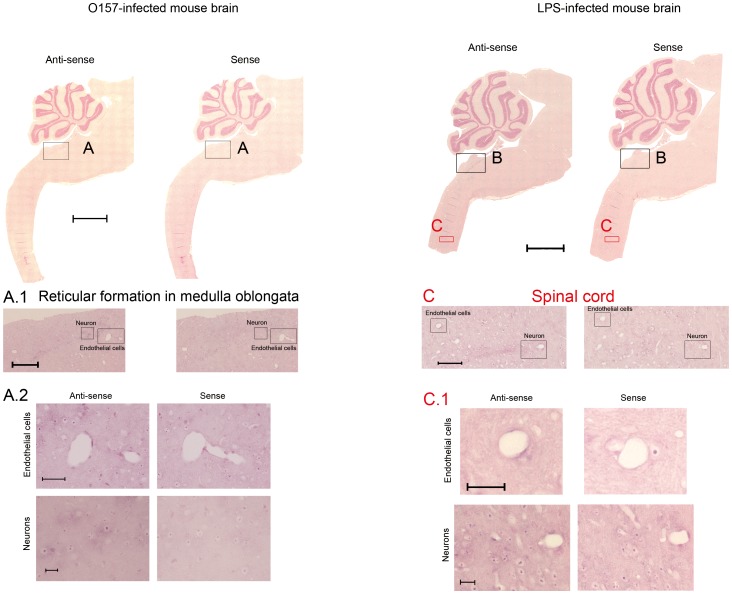
ISH of Gb3 synthase in brains of E32511 model and i.p.-injected LPS mice. (A) The brain of E32511-infected mice after hybridization of Gb3 synthase using Gb3syt anti-sense and sense probes. Scale bars: 1.5 mm. (A.1) Expression of Gb3 synthase was observed with anti-sense, but not sense probes in E32511-infected mice in the reticular formation of the medulla oblongata. Scale bars: 200 µm. (A.2) Magnification and expression of Gb3 synthase in endothelial cells (scale bars: 50 µm) and neurons (scale bars: 20 µm) of E32511-infected mouse brain, detected with anti-sense, but not sense probes. (B) The brain of i.p. LPS-injected mice after hybridization of Gb3 synthase using probe Gb3syt anti-sense and sense probes. Scale bars: 1.5 mm. (C) Expression of Gb3 synthase was observed in i.p. LPS-injected mice with the anti-sense, but not the sense probe in the spinal cord. Scale bars: 100 µm. (C.1) Magnification and expression of Gb3 synthase in endothelial cells (scale bars: 30 µm) and neurons (scale bars: 20 µm) of i.p. LPS-injected mice, detected with the anti-sense, but not the sense probe.

Three candidate probes for ISH are shown in (Table S1). To select the best probe for ISH, we did preliminary tests on mouse brain and we selected Gb3 synthase-1 (155–186) as the best probe. In our E32511 strain model, the anti-sense probes showed that Gb3 synthase mRNA was up-regulated in the reticular formation of the medulla oblongata (Figure 1A; Anti-sense). In the same region, Gb3 synthase mRNA was detected in ECs (Figure 1, A1 and A2; Anti-sense Endothelial cells) and neurons (Figure 1, A1 and A2; Anti-sense Neurons). As a positive control, LPS (500 µg/kg) was administered i.p. to the mice once a day for 3 consecutive days. The mouse brain was harvested on day 4 after three days of LPS administration. In the LPS-injected mouse model, we also detected Gb3 synthase mRNA in the reticular formation of the medulla oblongata using the anti-sense probes (Figure 1B; Anti-sense). Additionally, Gb3 synthase mRNA was up-regulated in the spinal cord (Figure 1C). In the spinal cord, Gb3 synthase mRNA expression was detected in ECs (Figure 1, C.1; Anti-sense Endothelial cells) and motor neurons in the anterior horn (Figure 1, C.1; Anti-sense Neurons). In the control mice, Gb3 synthase mRNA was not detected (Figure S1A). β-actin mRNA, as a positive control, was detected in the whole brain (Figure S1B). To the best of our knowledge, these are the first ISH data showing that Gb3 synthase mRNA is expressed in the ECs and neurons of the medulla oblongata and the anterior horn of the spinal cord in the brains of E32511 model and LPS-injected mice.

### Caspase-3 activation in the E32511 model

**Figure 2:**
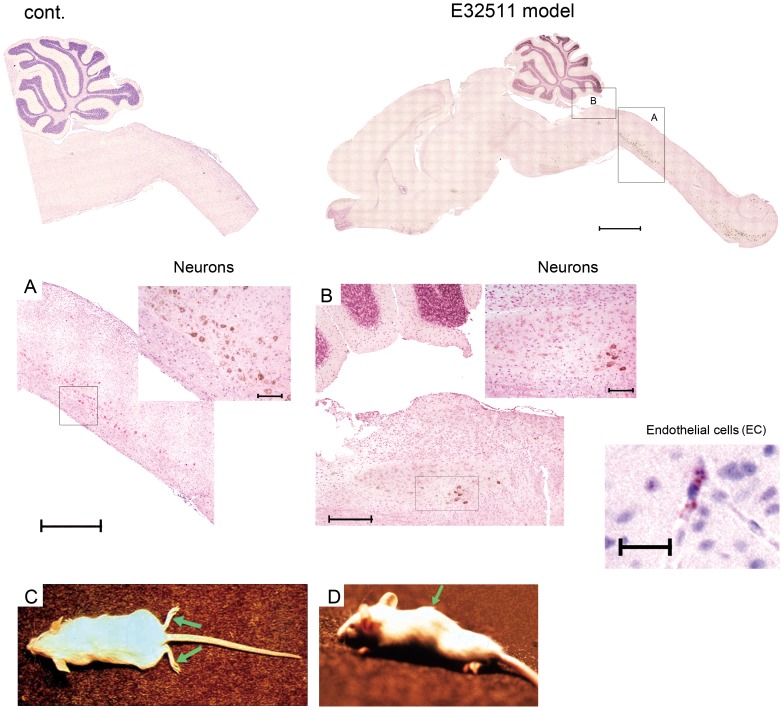
Caspase-3 activation in E32511 model mouse brain. Lesions were not detected in the brains of control (uninfected), whereas lesions were detected in the brains of E32511-infected. Scale bars: 1.5 mm. (A) Caspase-3 activation was detected in the neurons of the mouse spinal cord. Scale bars: 750 µm and 200 µm (inset). (B) Caspase-3 activation was also detected in neurons of the mouse reticular formation, scale bars: 300 µm and 200 µm (inset) and endothelial cells of the midbrain. Scale bars: 25 µm. (C) Four week-old female ICR outbred mice after inoculation. The representative clinical features of flaccid paralysis of the extremities were observed in ICR mice 3 to 5 days after orogastric challenge with E32511 10^11^ CFU/0.5 mL per mouse (green arrow). (D) Four week-old female ICR mice after inoculation. The representative clinical features of spinal deformity were observed in ICR mice 3 to 5 days after orogastric challenge with E32511/HSC 10^11^ CFU/0.5 mL per mouse (green arrow).

To investigate apoptosis in the affected mouse brain, we used immunohistochemistry to detect activated caspase-3. In the E32511 model, we detected caspase-3 activation in motor neurons in the anterior horn of the spinal cord (Figure 2A), in neurons in the reticular formation of the medulla oblongata (Figure 2B) and in ECs of the midbrain (Figure 2; EC). We also observed caspase-3 activation in the Stx2 (291 ng/kg)-injected mice in the neurons in the reticular formation of the medulla oblongata (Figure S2A), in motor neurons in the anterior horn of the spinal cord (Figure S2B), and in ECs in both areas (Figure S2A inset and S2B inset). After 5 days of E32511 inoculation, clinical symptoms were observed, such as paraplegia (Figures 2C and Video S1), spinal deformity (Figure 2D), and tremor, resulting to mice death within 24 h. Reconstruction of activated caspase-3 immunostaining clearly showed the 3-dimensional distribution of the apoptotic motor neurons in the anterior horn throughout the length of the spinal cord (Video S2, boxed part in Figure S2C). Overall, we detected up-regulated Gb3 synthase mRNA in the reticular formation of the medulla oblongata after oral infection with strain E32511, and in the anterior horn of the spinal cord after LPS stimulation. Caspase-3 activation was detected in the same locations as Gb3 synthase mRNA. Importantly, in the purified Stx2 i.p. injected mouse model, caspase-3 activation was also observed in the reticular formation of the medulla oblongata and the anterior horn of the spinal cord. Although we have no evidence of the Stx2 distribution in the mouse model, the ECs and neurons of the medulla oblongata and the anterior horn of the spinal cord might be the targets of Stx2c and Stx2. Caspase-3 activation was not detected in the mice injected i.p. with LPS (500 µg/kg) once a day for 3 consecutive days (data not shown).

### AST reactivity and diminished AQP4 expression in the E32511 model

**Figure 3:**
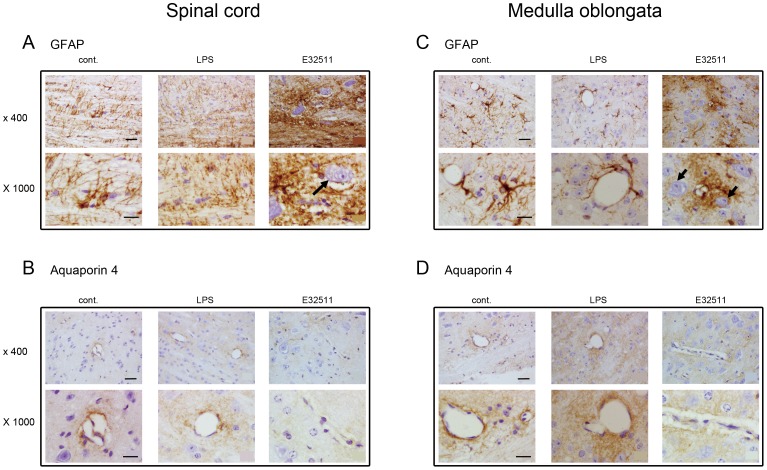
AST activation and AQP4 expression in E32511 model and i.p. LPS-injected mice. (A) Severe diffuse astrogliosis was observed in spinal cord of E32511-infected but not in control and LPS-injected mice at low (400×) and high (1000×) magnifications. The black arrow shows a degenerating neuron adjacent to an activated astrocyte. (B) More intense staining of AQP4 was observed surrounding the ECs which comprised the BBB in the spinal cord in control and LPS-injected mice, but not in E32511-infected mice at low (400×) and high (1000×) magnifications. (C) Severe diffuse astrogliosis was observed in the medulla oblongata of E32511-infected but not in control and LPS-injected mice at low (400×) and high (1000×) magnification. The black arrow shows a degenerating neuron adjacent to an activated astrocyte. (D) More intense staining of AQP4 was observed surrounding the blood vessels that comprise the BBB in the medulla oblongata in control and LPS-injected mice, but not in E32511-infected mice at low (400×) and high (1000×) magnifications. Scale bars (400×): 2 µm and (1000×): 1 µm.

We investigated the role of ASTs and the expression of AQP4 in the E32511 model using immunohistochemistry. GFAP immunoreactivity was stronger in the anterior horn of spinal cord of the E32511 model (Figure 3A; GFAP E32511) than in the control (Figure 3A; GFAP cont.) or the LPS-injected mice (Figure 3A; GFAP LPS). Around the reactive ASTs, the neurons in the spinal cord appeared to be degenerating, with vacuolar changes (Figure 3A; E32511, 1000×, arrow). In the reticular formation of the medulla oblongata, reactive ASTs with strong GFAP immunoreactivity were observed (Figure 3C; GFAP E32511) and the neurons around the reactive ASTs in the reticular formation of medulla oblongata also appeared to be degenerating, with vacuolar changes (Figure 3C; E32511, 1000×, arrow). In the control (Figure 3C; GFAP cont.) and LPS-injected mice (Figure 3C; GFAP LPS), astrocytic perivascular end-feet were observed (Figure 3C; GFAP cont. and LPS) and ASTs were not reactive, with normal end-feet (Figure 3C; GFAP cont. and LPS).

In the E32511 model, the expression of AQP4 decreased around the blood vessels in the anterior horn of the spinal cord (Figure 3B; E32511) and the reticular formation of the medulla oblongata (Figure 3D; E32511). On the other hand, a high density of AQP4 staining was observed in the perivascular areas in the anterior horn of the spinal cord and the reticular formation of the medulla oblongata (Figure 3B and D; cont. and LPS). Taken together, our results suggest that the damage to the BBB and neurons is associated with reactive ASTs. The down-regulation of AQP4 around the blood vessels may be part of the mechanism of BBB damage caused by reactive ASTs. Astrogliosis and the down-regulation of AQP4 around the blood vessels were not observed in the mice injected i.p. with LPS (500 µg/kg) once a day for 3 consecutive days (data not shown).

### Activation of microglia in the i.p. LPS mouse model and the E32511 model

**Figure 4:**
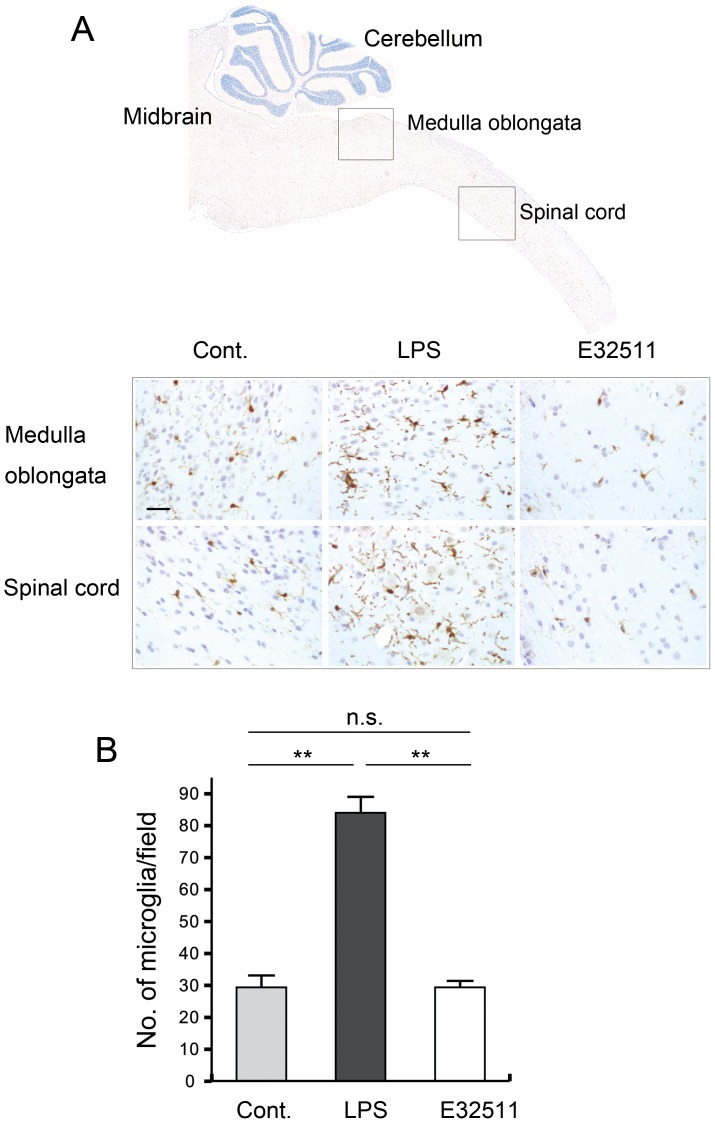
Microglial activation in i.p. LPS-injected and E32511 model mice. (A) Microglia in the medulla oblongata and the spinal cord were not activated in the control (uninfected) and E32511-infected mice, but were activated in i.p. LPS-injected mice. Scale bars: 2 µm. (B) The number of activated microglia significantly increased in the LPS-injected mice but not in the control and E32511-infected mice (LPS vs. control, *p*<0.0001; LPS vs. E32511, *p*<0.0001 and control vs. E32511, *p* = 1). Microglia were observed and counted manually from 5 non-overlapping fields. Statistical difference was measured by student's t test. **p<0.001.

In this experiment, we investigated whether microglia in the brain were activated in the E32511 model. Results revealed that the microglia in the medulla oblongata and the spinal cord of LPS-injected mice were activated (Figure 4A; LPS). Conversely, in the control (Figure 4A; cont.) and E32511-infected mice (Figure 4A; E32511), the microglia were not activated. Furthermore, to validate our observation, we counted the number of microglia from 5 non-overlapping fields in both the medulla oblongata and the spinal cord from each of the control, LPS and E32511 animal models. Results showed that there was a statistically significant increase in the number of microglia only in the i.p. injected LPS mice (*p*<0.0001). In the control and the E32511 model, microglias were not activated (Figure 4B; cont. and E32511). Taken together, our results indicate that microgliosis was not associated with the BBB and neurons damage caused by Stx2c in the E32511 model.

### The effects of AZM on encephalopathy in the E32511 model

**Figure 5:**
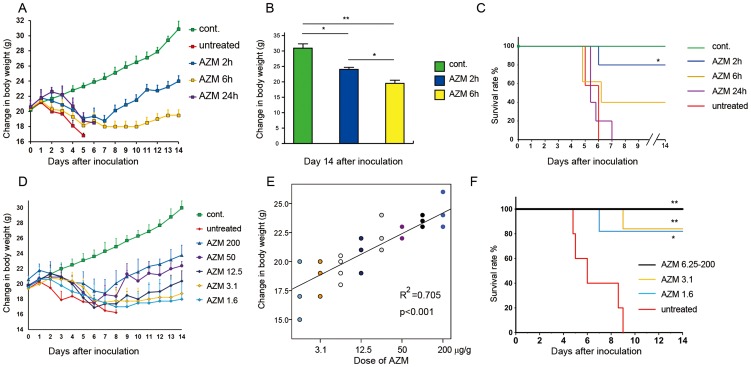
Effectiveness of a single dose of AZM in the E32511 model. (A) The administration of a single dose of AZM 2 h after infection significantly increased the BW of E32511-infected mice. (B) Mouse BW for AZM 2 h vs. AZM 6 h, *p* = 0.015 at day 14 after inoculation. Student's t test, *p<0.05; **p<0.001). (C) The administration of a single dose of AZM was significantly effective in the survival mice when given 2 h after infection, compared with 6 and 24 h after infection, (for AZM 2 h after infection vs. untreated, *p* = 0.012; AZM 2 h after infection vs. 24 h after infection, *p* = 0.013 and AZM 6 h and 24 h after infection vs. untreated, *p*>0.05. Log rank and χ^2^ test, (*p<0.05) (D) The administration of a single dose of 200–1.6 µg/g AZM 2 h after infection significantly increased the BW of E32511-infected mice. (E) The correlation between the dose of AZM and the increase in BW of E32511-infected mice was statistically significant, as shown by the Spearman's ρ (rho) = 0.887 and the *p* value, *p*<0.0001. Black-filled circle = AZM 100 µg/g; grey-filled circle = AZM 25 µg/g; empty circle = AZM 6.25 µg/g. (F) 1.6 µg/g AZM 2 h after infection with E32511 (10^11^ CFU/ mouse) was still effective in treating the E32511-infected mice, with a significant effect on the survival curve (200–6.25 µg/g AZM vs. untreated, *p* = 0.002; 3.1 µg/g AZM vs. untreated, *p* = 0.008 and 1.6 µg/g AZM vs. untreated, *p* = 0.016. Log rank and χ^2^ test, **p<0.01, *p<0.05.

Early treatment with a single dose of AZM (200 µg/g) 2 h after infection with E32511 at 1011 colony-forming unit (CFU) per mouse resulted in a significant increase in the BW of mice compared with those of the untreated group and the mice that were infected and treated 24 h later (p<0.05) (Figure 5A). Also at day 14 after infection, by Student's t-test, there was a significant increase in the BW of mice treated 2 h after infection compared with those treated 6 h after infection (p<0.05) (Figure 5B). We further observed that early treatment with a single dose of AZM (200 µg/g) 2 h after infection was very effective based on the survival of the infected mice (Figure 5C). To the best of our knowledge, these are the first data showing that an early treatment with a single dose of AZM (200 µg/g) is effective in treating the E32511 model and preventing encephalopathy. Subsequently, we investigated the effectiveness of AZM in a dose-dependent manner by applying a single dose of AZM 2 h after infection using a two-fold serial dilution of AZM (from 1.6 µg/g to 200 µg/g). In this experiment, mice lost weight after infection with E32511 at 10^11^ CFU per mouse, in an AZM dose-dependent manner (Figure 5D). By Spearman's test, there was a statistically significant correlation between the dose of AZM and the increase in the BW of the E32511 model mice (*p*<0.001) (Figure 5E). All of the mice treated with 3.1 µg/g to 200 µg/g of AZM survived compared with those infected with E32511 and untreated (*p*<0.001) (Figure 5F). Surprisingly, 80% of the mice treated with the minimum dose of AZM (1.6 µg/g) 2 h after infection survived (Figure 5F) (*p*<0.05). Histological examination of the spinal cord and the medulla oblongata revealed brain lesions in the infected but untreated mice; however, in the control and AZM-treated mice, no brain lesions were observed (Figures S3 and S4). Moreover, caspase-3 activation was not detected in the brains of mice treated with AZM (data not shown). Taken together, our results indicate that AZM is very effective in treating EHEC infection, especially in preventing encephalopathy in the E32511 model.

### The effects of AZM and other antimicrobial agents on encephalopathy in the E32511 model

**Figure 6:**
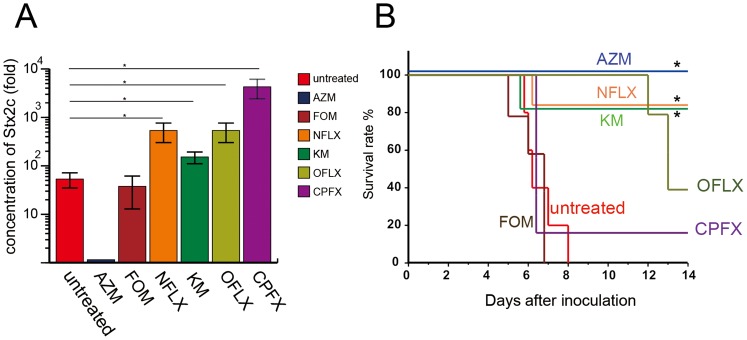
AZM compared with other antimicrobial agents in the E32511 model. (A) The concentration of Stx2c in NFLX, KM, OFLX, and CPFX treatment was significantly increased compared with control (no antibiotic) (NFLX vs. control, *p* = 0.023; KM vs. control, *p* = 0.02; OFLX vs. control, *p* = 0.023 and CPPFX vs. control, *p* = 0.017), while this was not the case for FOM. On the other hand, AZM statistically decreased Stx2c concentration compared with control (AZM vs. control, *p* = 0.007). Data were collected from the supernatants of cultures with 2-fold serial dilutions of each antimicrobial agent and are shown as mean and standard deviations of two independent experiments. Statistical difference was measured by Student's t test, *p<0.05. (B) AZM had a statistically significant effect on the mice survival, with 100% survival; NFLX and KM had a statistically significant effect on the mice survival, with 80% survival (AZM vs. untreated, *p* = 0.002; AZM vs. FOM, *p* = 0.003; AZM vs. CPFX, *p* = 0.014; AZM vs. NFLX and KM, *p*>0.05). Log Rank and χ^2^ test, *p<0.05.

We next compared the effectiveness of AZM with that of several antimicrobial agents recommended by the Japanese Government (including FOM, KM, and NFLX) in treating the E32511 model. The new quinolones, OFLX and CPFX, were also included in this experiment. The concentration of Stx2c in the culture supernatant was measured in the presence of sub-minimum inhibitory concentrations (MIC) (0.5× MIC) and the number of E32511 bacteria was counted before and after treatment with the antimicrobial agents including AZM (Table 1). Results revealed that the number of colonies that grew when treated with each microbial agent was almost the same. Results also showed that AZM did not induce the production of Stx2c. However, other antimicrobial agents induced the production of Stx2c (Figure 6A). FOM, NLFX, KM, OFLX, and CPFX increased Stx2c production to 1.5-, 12.5-, 2.25-, 12.5- and 100-fold of control (without the antimicrobial agents), respectively (Figure 6A and Table 1). The concentration of antimicrobials was adjusted to 25× MIC and the mice were treated with a single dose at this concentration 2 h after infection with the E32511 strain. The results revealed that AZM-treated mice had a 100% survival rate, which was higher significantly compared to the untreated group (0% survival rate) (*p*<0.01). KM and NFLX-treated mice, on the other hand, showed an 80% survival rate, which was also significantly higher compared to the untreated group (*p*<0.05) (Figure 6B). All the FOM-treated mice died, and there was no statistically significant difference between the survival rate of CPFX- or OFLX-treated mice, and untreated mice (Figure 6B). The MIC of CPFX and OFLX were 0.2 and 0.03 µg/mL, respectively (Table 1), and these new quinolones were shown to have strong bactericidal effects. We treated the infected mice with high doses of CPFX and OFLX, 333× and 75× MIC, respectively. Results showed that there was a statistically significant difference in the survival rate between mice treated with these high doses and the untreated mice (Figure S5). However, the survival rates of CPFX- and OFLX-treated mice remained at 40% and 30%, respectively. From these results, we suggest that a single dose of AZM (200 µg/g) given at an early stage provides more favorable results than other antimicrobial agents. Additionally, AZM-treated mice survived due in part to a lower production of Stx2c than in the untreated mice.

**Table 1:** Bacterial count at sub-minimum inhibitory concentration (MIC) of AZM and other antimicrobial agents against E32511. AZM, azithromycin; FOM, fosfomycin; NFLX, norfloxacin; KM, kanamycin; OFLX, orfloxacin; CPFX, ciprofloxacin.

Antimicrobial agents	MIC (µg/mL)	Colony count (0 h) (CFU/mL)	Colony count at 18 h after antimicrobial (0.5xMIC)	Concentration of Stx2c (folds) added 0.5×MIC	Per os dose of antimicrobial to mice (25×MIC) µg/gram	Survival rate (%) of mice
(-)	0	1.0×10^5^	1.18±0.23×10^9^	48±23	0	0
AZM	8		3.37±0.15×10^8^	0	200	100
FOM	1.5		1.37±0.23×10^8^	40±34	39	0
NFLX	0.2		2.63±0.15×10^7^	600±283	313	80
KM	12.5		1.17±0.12×10^9^	164±51	4.9	80
OFLX	0.2		3.22±0.11×10^7^	600±51	5.0	40
CPFX	0.03		2.52±0.23×10^5^	4800±2263	0.75	20

AZM, azithromycin; FOM, fosfomycin; NFLX, norfloxacin; KM, kanamycin; OFLX, orfloxacin; CPFX, ciprofloxacin.

## Discussion

In this study we report five significant results. 1) Using ISH, we detected Gb3 synthase mRNA in the neurons and ECs of the anterior horn of the spinal cord and the reticular formation of the medulla oblongata of infected mice. Importantly, no Gb3 synthase mRNA expression was detected in the control mice. In the E32511 model, Gb3 synthase mRNA was up-regulated in the reticular formation of the medulla oblongata due to bacterial infection-induced brain Gb3 synthesis and generated toxin receptors. In the LPS-injected mouse model, Gb3 synthase mRNA was induced in both the reticular formation of the medulla oblongata and the spinal cord. 2) In the E32511 model, caspase-3 activation was detected in neurons and ECs of the anterior horn of the spinal cord and the reticular formation of the medulla oblongata. 3) GFAP immunostaining revealed that the ASTs surrounding the apoptotic neurons of the anterior horn of spinal cord were activated in the E325211-infected mice, but not in the control and LPS-injected mice. Also, in the reticular formation of the medulla oblongata, perivascular cytoplasmic astrogliosis was observed with a high intensity of GFAP immunostaining. 4) In the E32511 model, perivascular AQP4 could not be detected in the anterior horn of the spinal cord and the reticular formation of the medulla oblongata. On the other hand, AQP4 was strongly expressed in the AST vascular foot processes in control and LPS-injected mice. The reduced AQP4 expression provided evidence of BBB impairment. 5) The macrolide antibiotic AZM was strongly effective in the E32511 model infected with 10^11^ CFU of E32511.

The receptor for Stxs is synthesized from lactosylceramide by Gb3 synthase [35]. In a previous report, Gb3 synthase^−/−^ mice survived after intravenous injection of Stx1 and Stx2 [36]. Gb3 was found to be expressed in the microvascular ECs of the brain using immunohistochemistry [36]. Obata *et al*. also reported that Gb3 was localized in neurons of mouse brain, and in both neurons and ECs in human brain [25]. In our study, we found that the pro-inflammatory response up-regulated Gb3 synthase mRNA in neurons and ECs of E32511-infected mice, but not in control mouse brain. The high Gb3 expression in the brain associated with a pro-inflammatory response may be a risk factor for neurological impairment in EHEC infection. Importantly, up-regulated Gb3 was found to be localized in the anterior horn of the spinal cord and the reticular formation of the medulla oblongata in our model. Many apoptotic neurons were also observed in the same area. We previously reported that MRI showed high-intensity areas in the spinal cord, brain stem, and medulla oblongata 48 h after i.v. administration of purified Stx2 in rabbits, and that the cause of death might be circulatory failure caused by impairment of the cardiovascular centre in the medulla oblongata [11]. In the E32511 model, E32511-infected mice developed paralysis of the upper and lower extremities (Video S1) and deformity of the spinal cord, which were thought to be due to apoptosis of the motor neurons in the anterior horn of the cervical spinal cord (Video S2). Apoptosis of motor neurons in the anterior horn of the spinal cord that control respiratory function might be the cause of death in the E32511 model.

In the EHEC O104:H4 outbreak, post-mortem neuropathological examination of the patients who developed severe neurological symptoms showed that astrogliosis were most prominent around the blood vessels, and microgliosis was diffusely observed in the thalamus and pons in human brain [37]. Golstain *et al*. reported that there was a high incidence of injured ASTs among those that form the BBB with ECs in rats that received intracerebroventricular administration of Stx2 [38]. The role of ASTs or microglia in EHEC infection is still unknown. Recently, AST-derived factors such as TNF-α, nitric oxide, and chemokines were found to alter the permeability of brain ECs and promote the adhesion of polymorphonuclear neutrophils and platelets in an *in vitro* study [26].

In our E32511 model, we observed GFAP-positive reactive ASTs around blood vessels in the anterior horn of the spinal cord and the reticular formation of the medulla oblongata. The reduction in AQP4 expression around blood vessels suggested that the integrity of BBB was compromised either by a direct effect of Stx2c or by reactive ASTs. However, microglias were not associated with the brain damage caused by E32511. In a previous report, rats injected i.v. with Stx2 supernatant were shown to have significantly decreased expression levels of AQP1 and AQP4 in the brain [27]. In the freeze-injury model of vasogenic brain edema, AQP4-deficient mice had the worst clinical outcome [39]. It was also reported that AQP4-null mice had aggravated vasogenic edema [40]. In our study, we showed that inflammatory stimuli caused by E32511 infection or i.p. injected LPS increased Gb3 synthase mRNA in ECs and neurons in the anterior horn of the spinal cord and the reticular formation of the medulla oblongata. The role of BBB damage is unclear in the Stx toxemia animal model. Reactive ASTs affected the integrity of the BBB and polymorphonuclear neutrophil (PMN)-mediated endothelial toxicity [41]. Also, ASTs mediated the maintenance of the BBB [42] and removal of ASTs from co-culture with ECs resulted in increased permeability [43]. Reactive ASTs can release immune mediators that may exert neurotoxic effects [44]. Notably, B cell-activating factor (BAFF), from the TNF superfamily, is strongly up-regulated in reactive ASTs in the demyelinated lesions of multiple sclerosis [45]. In the E32511 model, Stx2 was detected in destroyed nerve fibers whose axons were found to be edematous and vacuolated [8]. The destroyed nerve fibers might be associated with BAFF released by the reactive ASTs. It has been reported that nerve growth factor secreted by reactive ASTs induces motor neuron apoptosis through a p75NTR-dependent mechanism [46]. In the present study, the activation mechanism of the ASTs still remains unclear, but the reactive ASTs could amplify the BBB damage in the E32511 model. We propose three possibilities for the activation mechanism of ASTs. First, the ASTs could be directly activated by Stx2c, because Stx2c was detected in mouse brain parenchyma in ECs and nerve fibers using labeled Stx2c in an immunoelectron-microscopic study [8]. The reactive ASTs can then release neurotoxic factors and EC-toxic factors, such as TNFα, nitric oxide, IL-6, and IL-1β resulting in damage to the neurons and the BBB. Second, Stx2c absorbed from the mouse intestine binds to the up-regulated Gb3 of the ECs and causes EC apoptosis. The leakage of Stx2c through the BBB then causes neuronal apoptosis. Third, the apoptotic neurons activate the ASTs, and the reactive ASTs would be unable to support BBB integrity and would cause the down-regulation of AQP4. Also, the down-regulation of AQP4 could amplify a signaling loop associated with vasogenic brain edema.

Recently, Zhang *et al*. reported that a pediatric dose of AZM was effective in the piglet model orally infected with Stx2-producing *E. coli*, and that *rec A* mutants treated with the phage-inducing agent MMC and a sub-inhibitory concentration of CPFX failed to produce Stx2 [47]. In this study, we investigated the effectiveness of AZM against the E32511 model infected with 10^11^ CFU of E32511 and given an i.p. injection of MMC. The inoculating dose of E32511 in this model was modified from 10^10^ CFU to 10^11^ CFU and resulted in 100% death, which was higher from the previously observed 90% death rate [8]. Based on the results of treatment with AZM, the administration of a single 200 µg/g dose of AZM 2 h after inoculation of bacteria in the E32511 model was shown to be significantly effective in improving the survival of infected mice, compared with those treated either 6 or 24 h after inoculation. Ikeda *et al*. reported that the early use of FOM, within the first two days of diarrhea, prevented the development of HUS [28]. However, FOM therapy on and after the third day of diarrhea could not prevent HUS. In the E32511 model, MMC might cause a high production of Stx2c from bacteria 6 h after inoculation with strain E32511. Before the high production of Stx2c induced by MMC phage induction, AZM might inhibit the release of the toxin. Apart from these direct antimicrobial effects, AZM has also been reported to function as an immunomodulator to suppress the production of inflammatory cytokines such as TNF-α, IL-1β, and IL-6 by Stx1 [31]. Intraperitoneal injection of AZM could protect the immature mice inoculated with 10^9^ CFU/mice orally followed by 3 days treatment with a high amount of AZM (10 mg/kg) [31]. In our result, we revealed that oral treatment of AZM could protect the infected mouse model by inhibiting the production of Shiga toxin 2 using a single lower dose (1.6 mg/kg). AZM showed a 100% survival rate, while NLFX and KM showed an 80% survival rate, and FOM showed no significant effect on the survival rate. These results are in congruence with our *in vitro* results, in which only AZM did not induce the production of Stx2c, while the other antimicrobial agents induced the release of Stx2c. The addition of antimicrobial agents other than AZM induced higher Stx2c production than that observed in the positive control without antimicrobial agents. Antibiotic use during EHEC O157:H7 infections has been reported to be associated with a higher rate of subsequent HUS and should be avoided [48]. However, in the biggest outbreak of EHEC O104:H4 in Germany, treatment with AZM was associated with a lower frequency of long-term STEC carrier status [49].

To investigate encephalopathy caused by EHEC infection and i.p.-injected LPS, we first succeeded in detecting Gb3 synthase mRNA by ISH. High Gb3 synthase mRNA expression was found in the ECs and neurons in the anterior horn of the spinal cord and the reticular formation of the medulla oblongata. To understand the mechanism of BBB damage in our mouse model inoculated with 10^11^ CFU of E32511, we focused on the pro-inflammatory response caused by Stx2c-producing EHEC. The pro-inflammatory response induced up-regulation of Gb3 synthase mRNA in ECs and neurons, activation of ASTs, and a decrease in AQP4. In conclusion, apoptosis of the neurons in the spinal cord and in the medulla oblongata, together with respiratory and autonomic dysfunction, may be the cause of death in our mouse model.

## Supporting Information

Figure S1 (A) Sagittal sections of the whole brain of control mice hybridized with anti-sense and sense Gb3 synthase probes. (B) Sagittal sections of the whole brain of E32511-infected and LPS administered intraperitoneally mice hybridized with anti-sense β-actin probe. Scale bars: 3 mm.
(TIF)

Figure S2 **Caspase-3 activation in mouse brain injected with Stx2.** Whole brain of i.p. Stx2-injected mice. Scale bars: 3 mm. (**A**) Caspase-3 activation was detected in the neurons of the reticular formation of the medulla oblongata. Scale bars: 200 mm. (**B**) Caspase-3 activation was detected in the neurons of the anterior horn of the spinal cord. (**C**) The orientations of 3D images of the mouse spinal cord (Video S2) is shown in the boxed part of C.
(TIF)

Figure S3 **Structural features of histological changes in the spinal cord of the E32511 model.** Sections of the spinal cord from control (uninfected) mice (**A, D, G**), AZM-untreated mice (**B, E, H**) and AZM-treated mice (**C, F, I**) stained with H&E are shown. **A–C** with original 100× magnification; **D–F** with 400× magnification and **G–I** with 1000× magnification. Intact motor neurons were observed in control (uninfected) mice: **A, D, G** and AZM-treated mice: **C, F, I**. Degenerating motor neurons with vacuolar changes (arrow in **H**) were observed in AZM-untreated mice: **B, E, H**. Scale bars: A: 10 µm; D: 2 µm; G: 1 µm.
(TIF)

Figure S4 **Structural features of histological changes in the medulla oblongata of the E32511 model.** Sections of brain stems (medulla oblongata) from control (uninfected) mice (**A, D, G**), AZM-untreated mice (**B, E, H**) and AZM-treated mice (**C, F, I**) stained with H&E are shown. **A–C** with original 100× magnification; **D–F** with 400× magnification; and **G–I** with 1000× magnification. Intact neurons were observed in control (uninfected) mice: **A, D, G** and AZM-treated mice: **C, F, I**. Degenerating neurons with vacuolar changes (arrows in **E** and **H**) were observed in the reticular formation in AZM-untreated mice: **B, E, H**. Scale bars: A: 10 µm; D: 2 µm; G: 1 µm.
(TIF)

Figure S5 **Clinical dose of CPFX and OFLX in the E32511 model.** 10 µg/g CPFX 10 and 15 µg/g OFLX 15 had a statistically significant effect on the mice survival; however, CPFX and OFLX treatments were not very effective, with 40% and 30% survival, respectively (CPFX vs. untreated, *p*<0.0001; OFLX vs. untreated, *p* = 0.013. Log Rank and χ^2^ test, *p<0.05, **p<0.001.
(TIF)

Table S1 **Sequence of probes used in** ***in situ*** **hybridization.**
(PDF)

Video S1 **Video of the mouse showing symptoms after infection with E32511 1×10^11^ CFU/0.5 mL.** The symptoms developed from 3 to 5 days after E32511 infection. The symptoms of tremor, paralysis of hind extremities, and spinal deformity were observed in infected, but not uninfected mice.
(MP4)

Video S2 **The orientation of 3D images of the mouse spinal cord is shown in the boxed part of A in Figure S2C.** Apoptotic motor neurons are indicated in the anterior horn of the spinal cord of mice infected with EHEC O 157 :H- Strain E32511 1×10^11^ CFU.
(MP4)
