# Supplementary figures and images for: Investigation of Encephalopathy Caused by Shiga Toxin 2c-Producing Escherichia coli Infection in Mice
Source: PLoS One. 2013 Mar 13;8(3):e58959. doi: 10.1371/journal.pone.0058959 (PMC3596349; doi:10.1371/journal.pone.0058959)

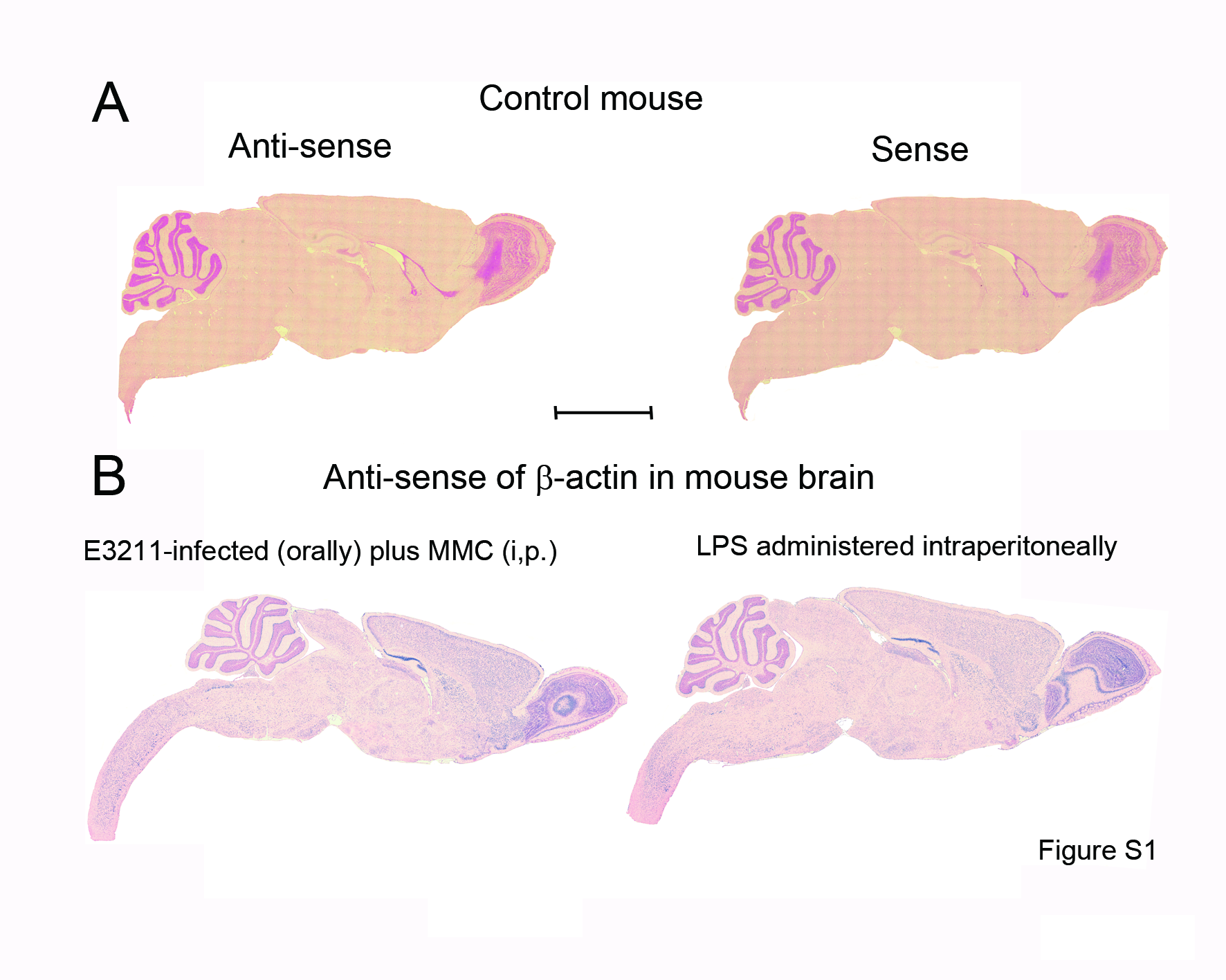

Supplement: Figure S1 — (A) Sagittal sections of the whole brain of control mice hybridized with anti-sense and sense Gb3 synthase probes. (B) Sagittal sections of the whole brain of E32511-infected and LPS administered intraperitoneally mice hybridized with anti-sense β-actin probe. Scale bars: 3 mm. (TIF) [file pone.0058959.s001.tif]

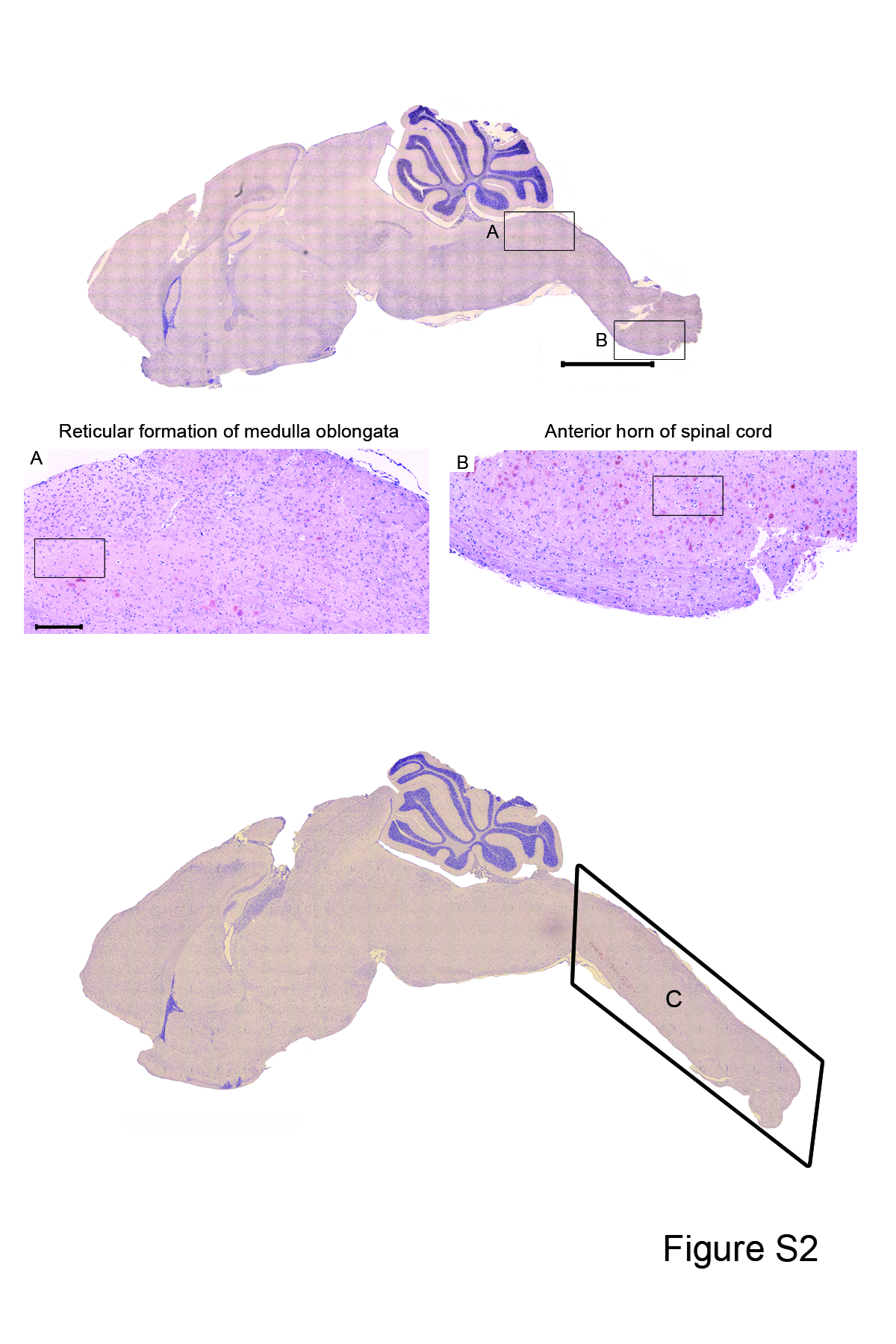

Supplement: Figure S2 — Caspase-3 activation in mouse brain injected with Stx2. Whole brain of i.p. Stx2-injected mice. Scale bars: 3 mm. (A) Caspase-3 activation was detected in the neurons of the reticular formation of the medulla oblongata. Scale bars: 200 mm. (B) Caspase-3 activation was detected in the neurons of the anterior horn of the spinal cord. (C) The orientations of 3D images of the mouse spinal cord (Video S2) is shown in the boxed part of C. (TIF) [file pone.0058959.s002.tif]

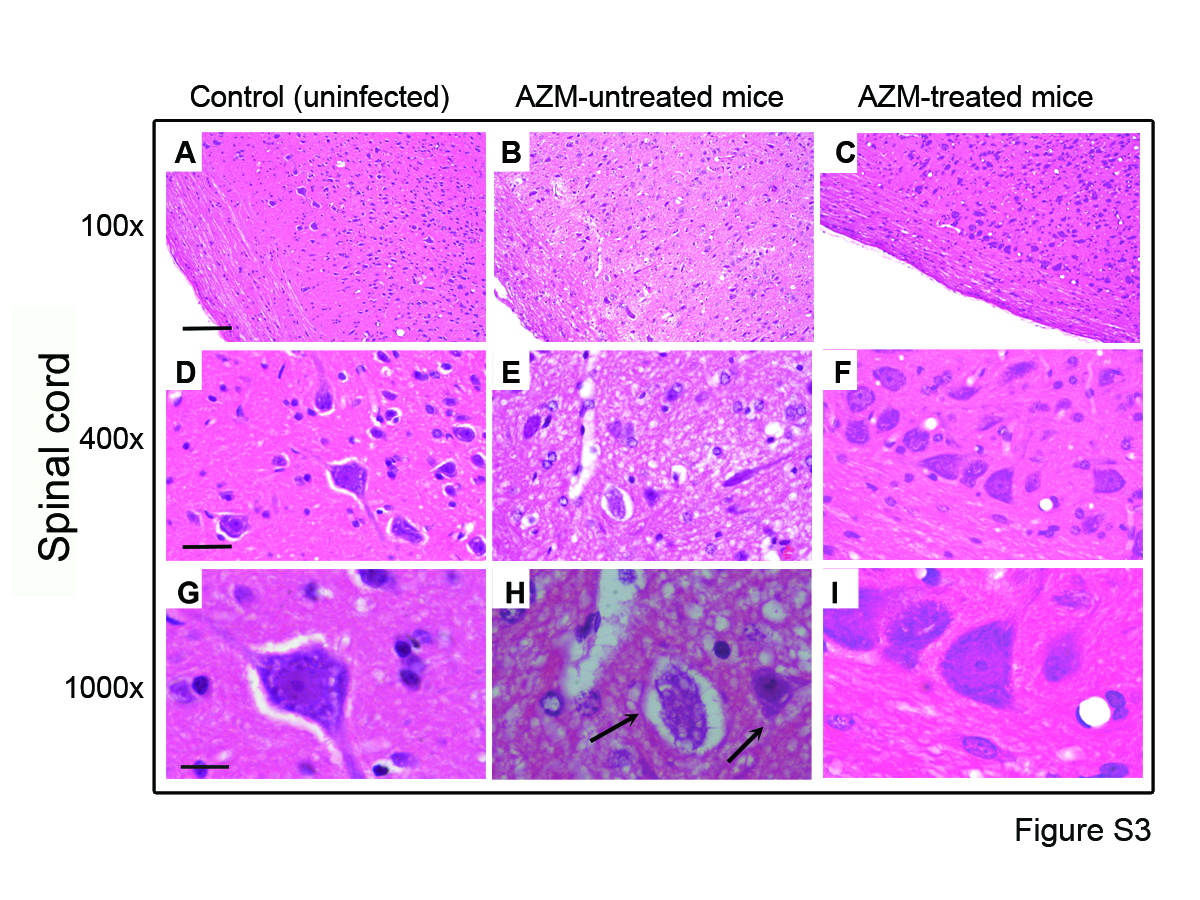

Supplement: Figure S3 — Structural features of histological changes in the spinal cord of the E32511 model. Sections of the spinal cord from control (uninfected) mice (A, D, G), AZM-untreated mice (B, E, H) and AZM-treated mice (C, F, I) stained with H&E are shown. A–C with original 100× magnification; D–F with 400× magnification and G–I with 1000× magnification. Intact motor neurons were observed in control (uninfected) mice: A, D, G and AZM-treated mice: C, F, I. Degenerating motor neurons with vacuolar changes (arrow in H) were observed in AZM-untreated mice: B, E, H. Scale bars: A: 10 µm; D: 2 µm; G: 1 µm. (TIF) [file pone.0058959.s003.tif]

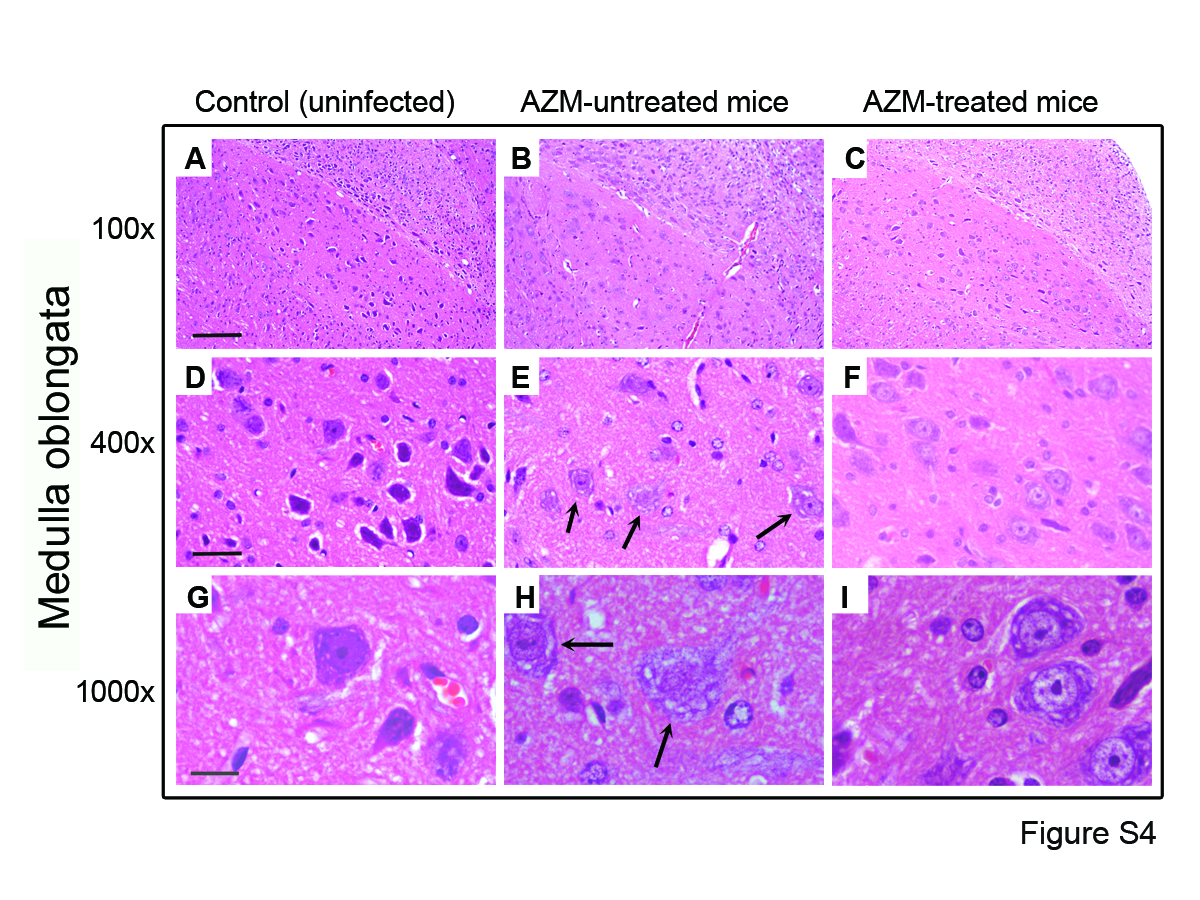

Supplement: Figure S4 — Structural features of histological changes in the medulla oblongata of the E32511 model. Sections of brain stems (medulla oblongata) from control (uninfected) mice (A, D, G), AZM-untreated mice (B, E, H) and AZM-treated mice (C, F, I) stained with H&E are shown. A–C with original 100× magnification; D–F with 400× magnification; and G–I with 1000× magnification. Intact neurons were observed in control (uninfected) mice: A, D, G and AZM-treated mice: C, F, I. Degenerating neurons with vacuolar changes (arrows in E and H) were observed in the reticular formation in AZM-untreated mice: B, E, H. Scale bars: A: 10 µm; D: 2 µm; G: 1 µm. (TIF) [file pone.0058959.s004.tif]

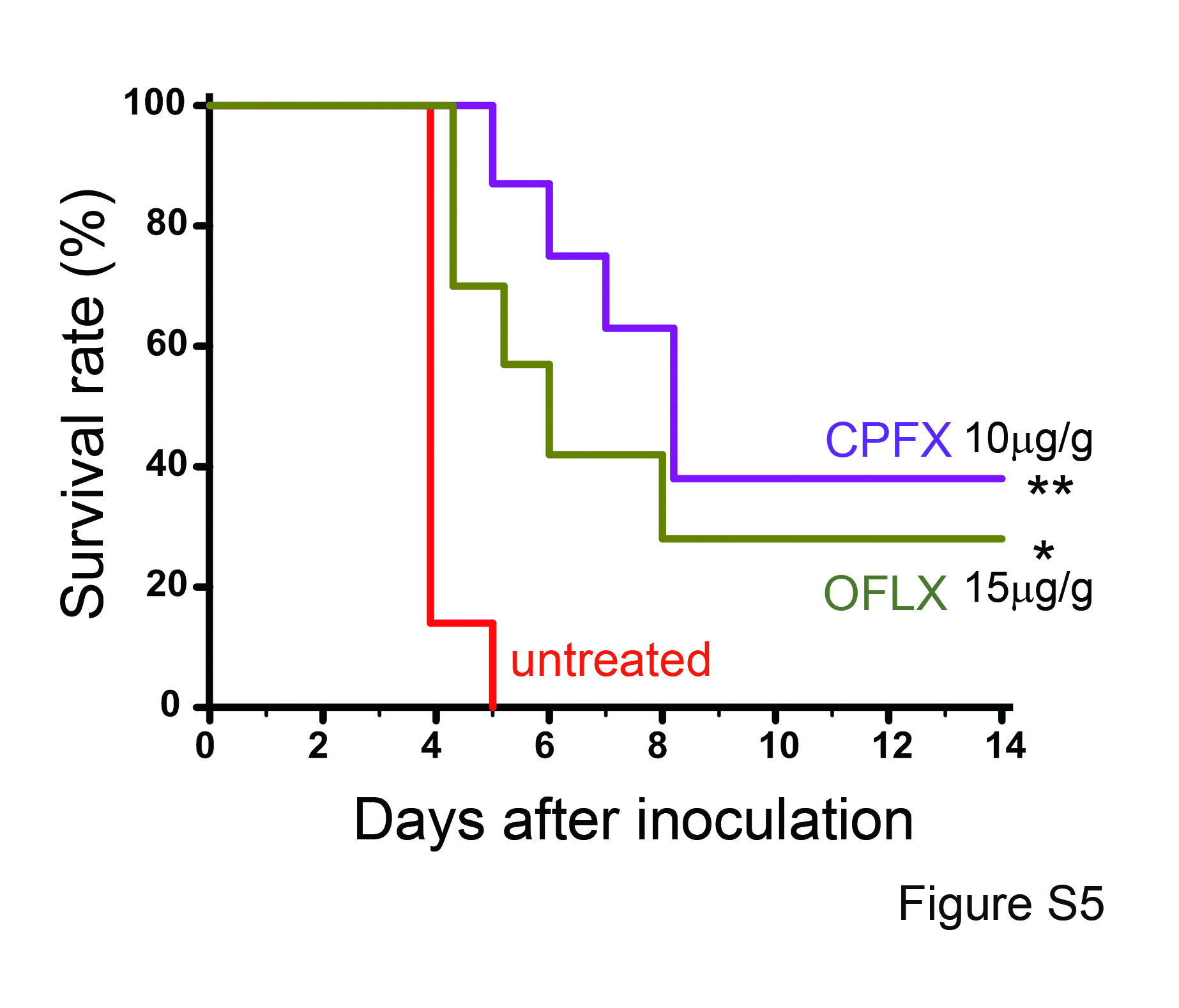

Supplement: Figure S5 — Clinical dose of CPFX and OFLX in the E32511 model. 10 µg/g CPFX 10 and 15 µg/g OFLX 15 had a statistically significant effect on the mice survival; however, CPFX and OFLX treatments were not very effective, with 40% and 30% survival, respectively (CPFX vs. untreated, p<0.0001; OFLX vs. untreated, p = 0.013. Log Rank and χ2 test, *p<0.05, **p<0.001. (TIF) [file pone.0058959.s005.tif]
