# Supplementary material for: Investigation of Encephalopathy Caused by Shiga Toxin 2c-Producing Escherichia coli Infection in Mice
Source: PLoS One. 2013 Mar 13;8(3):e58959. doi: 10.1371/journal.pone.0058959 (PMC3596349; doi:10.1371/journal.pone.0058959)
Supplement: Table S1 — Sequence of probes used in in situ hybridization. (PDF) [file pone.0058959.s006.pdf]

Table S1. Sequence of probes used in *in situ* hybridization

Probe	Sequence position	Sequence size	Sequence
**Gb3syt-1**	155-686	532	TACTCCGGTTGGCACAGAAGACTAGTTCCTAACCCAGTGTGCATGTCCAGGATGCCTGCAGGCTGCTAGGACCCCTCACCTCCTTGTTATGGACTGCCCAGGCTGACAAGTCAATCTTTATGGGCATCTCCTGTTCCCATCTGGAGGAGACCATGTCCAAGCCCCCCGACTGCCTGCTGCGGATGCTCAGGGGCACCCCCAGACAGCGGGTCTTCACCTTCTTCATCATCAGCTTCAAGTTCATGTTCTTGATCTCCATCCTGATCTACTGGCACACTGTGGGTGCACCCAAGGACCAAAGAGAGTATAGCCTGCCAGTAGACTTCTCCTGCCCCCAGCTGGCCTTTCCTAGAGTCTCTGCCCCAGGCAACATCTTCTTCCTAGAGACATCGGACAGGACCAGTCCCAACTTTCTATTTATGTGTTCTGTGGAATCAGCTGCCAGGGCACACCCTGAATCCCAGGTGGTTGTGCTGATGAAAGGGCTGCCTAGGGACACCACAGCTCAGCCCCGGAACCTCGGCATCTCTCT
**Gb3syt-2**	707-1199	493	TCTGGATACGACCTCTGGACTTGCAAGAACTGTTTGAGGACACACCACTGGCAGCCTGGTACTCGGAGGCACGGCACAGATGGGAGCCCTACCAGTTGCCCGTACTGTCTGATGCTTCCAGGATCGCACTCCTCTGGAAGTTTGGTGGGATCTACCTGGACACAGACTTCATCGTCCTCAAGAACCTGCTCAACCTGACCAACACGCTGGGCATCCAATCCCGCTATGTCCTCAACGGTGCCTTCCTAGCCTTCGAGCGCAAGCATGAGTTCTTGGCACTGTGTCTACATGACTTCGTGGCCAACTACAATGGTTGGATTTGGGGTCACCAGGGCCCCCAACTGCTTACCCGAGTCTTCAAGAAGTGGTGTTCCATCCAAAGCCTGGAAAAGAGCCATGCTTGCCGTGGGGTCACTGCCCTGCCCCCCGAGGCCTTCTACCCCATCCCCTGGCAGAACTGGAAGAAATACTTTGAAGACATCAGCCCCGAGGA
**Gb3syt-3**	1210-1677	468	CTGCTCAATGCCACCTATGCTGTTCATGTGTGGAACAAGAAAAGTCAGGGTACACACCTAGAGGCCACATCCAAGGCTCTGCTGGCCCAGCTACATGCCCGCTATTGCCCTACGACACATAGAGCCATGAAGATGTACTTGTGACAGCCCTTCAGGCTGCAGTCATATCATATCAACATTGTTACCGCCTTTCCAGGGAGACAAGATGAGCAGACAGGGAAAAAGGGGCCTTTGAGATGCTGCTGTTGACTTTAGAGGAGACCAAGCTGGCCTGGGGAACACTTGCCAGTTCTACACCTGCCCCTCTAAGCCAAAGGGAGACCGATGGAGAACACTTTGTCCGGTGTTCTAGGCTGACCAGTGATTACAGAATTACTAACATCTGGGGCCTTAATGGGCTAGATCACAAGAACACTAAAGAGCATTCTAAGGTAGCTGGAAGACAGCTGGTTAGAGGTGGGGTCAGGA
